# Antimalarial Drug Resistance: Literature Review and Activities and Findings of the ICEMR Network

**DOI:** 10.4269/ajtmh.15-0007

**Published:** 2015-09-02

**Authors:** Liwang Cui, Sungano Mharakurwa, Daouda Ndiaye, Pradipsinh K. Rathod, Philip J. Rosenthal

**Affiliations:** Department of Entomology, Pennsylvania State University, University Park, Pennsylvania; Malaria Research Department, Macha Research Trust, Johns Hopkins Malaria Research Institute, Choma, Zambia; Department of Parasitology and Mycology, Cheikh Anta Diop University, Dakar, Senegal; Department of Chemistry, University of Washington, Seattle, Washington; Department of Medicine, University of California, San Francisco, San Francisco, California

## Abstract

Antimalarial drugs are key tools for the control and elimination of malaria. Recent decreases in the global malaria burden are likely due, in part, to the deployment of artemisinin-based combination therapies. Therefore, the emergence and potential spread of artemisinin-resistant parasites in southeast Asia and changes in sensitivities to artemisinin partner drugs have raised concerns. In recognition of this urgent threat, the International Centers of Excellence for Malaria Research (ICEMRs) are closely monitoring antimalarial drug efficacy and studying the mechanisms underlying drug resistance. At multiple sentinel sites of the global ICEMR network, research activities include clinical studies to track the efficacies of antimalarial drugs, ex vivo/in vitro assays to measure drug susceptibilities of parasite isolates, and characterization of resistance-mediating parasite polymorphisms. Taken together, these efforts offer an increasingly comprehensive assessment of the efficacies of antimalarial therapies, and enable us to predict the emergence of drug resistance and to guide local antimalarial drug policies. Here we briefly review worldwide antimalarial drug resistance concerns, summarize research activities of the ICEMRs related to drug resistance, and assess the global impacts of the ICEMR programs.

## Introduction

Despite important gains in some areas, malaria remains a major problem in most of the tropical world, and it continues to cause hundreds of millions of illnesses and hundreds of thousands of deaths each year.^1^ Most serious illnesses and deaths from malaria and also most drug-resistant infections are due to infection with *Plasmodium falciparum*, the most virulent human malaria parasite. In addition, there is increasing appreciation of the importance of *Plasmodium vivax*, the other common human malaria parasite, as a cause of serious illnesses, and its resistance to antimalarial drugs is now well described.^2^

The control and eventual eradication of malaria depend on a small set of tools. For control of anopheline mosquito vectors the values of insecticide-impregnated bednets and indoor residual spraying of insecticides have been clearly demonstrated,^3^ but their efficacy will be limited without coincident efforts directed against malaria parasites. An effective vaccine against malaria would be extremely valuable. Unfortunately, although the RTS,S vaccine, which has offered modest protection against malaria in African children,^4^ may be available in a few years, no highly effective vaccine is on the horizon. Thus, appropriate use of antimalarial drugs remains a cornerstone of malaria control. Drugs have two key roles for malaria control. First, prompt and effective treatment of malaria prevents progression to severe disease and limits the development of gametocytes, thus blocking transmission to mosquitoes.^5^ Second, drugs can be used to prevent malaria in endemic populations, including various strategies of chemoprophylaxis, intermittent preventive therapy, and mass drug administration.^6^

The ICEMR network includes 10 groups focused on malaria surveillance and related activities in 10 different malaria-endemic regions. This article discusses current knowledge of antimalarial drug resistance, including activities of ICEMR groups to assess and characterize resistance in different regions.

## Antimalarial Drugs

Antimalarial drugs act principally to eliminate the erythrocytic stages of malaria parasites that are responsible for human illness. Drug regimens for treatment of the two most prevalent malaria parasites, *P*. *falciparum* and *P*. *vivax*, are different. With frequent resistance to older drugs, artemisinin-based combination therapy (ACT) is now recommended for the treatment of uncomplicated falciparum malaria in nearly all areas.^7^ Chloroquine plus primaquine remains the first-line regimen for radical cure of vivax malaria in most regions. ACT consists of a potent artemisinin component, which rapidly clears most parasites, plus a longer acting partner drug, which eliminates remaining parasites and limits selection of artemisinin resistance.^7^ The ACTs recommended by the World Health Organization (WHO) are artemether/lumefantrine, artesunate/amodiaquine, artesunate/mefloquine, dihydroartemisinin/piperaquine, artesunate/pyronaridine, and artesunate/sulfadoxine–pyrimethamine. ACTs are also effective against erythrocytic stages of non-falciparum malaria parasites. Multiple drugs are used to prevent malaria. Recommendations for travelers from nonendemic to endemic areas generally advocate use of atovaquone/proguanil (Malarone), mefloquine, or doxycycline in chemoprophylactic regimens.^8^ In Africa, intermittent preventive therapy is advocated in some high-risk populations, including sulfadoxine/pyrimethamine during pregnancy and amodiaquine/sulfadoxine–pyrimethamine as seasonal malaria chemoprophylaxis in the Sahel subregion, where there is little resistance to these drugs.^6^

Available antimalarial drugs can be divided into multiple classes (Table 1). The 4-aminoquinoline, chloroquine, was the gold standard for the treatment of uncomplicated malaria for many years, but it is no longer appropriate for the treatment of falciparum malaria in nearly all areas because of drug resistance. In Indonesia, increased resistance to chloroquine in *P*. *vivax* prompted a policy change to ACTs for vivax malaria.^9^ Amodiaquine appears to be subject to the same resistance mechanisms as chloroquine, but due to improved potency it provides adequate efficacy against many chloroquine-resistant parasites, and it is a component of the widely used ACT artesunate/amodiaquine. A third 4-aminoquinoline, piperaquine, was widely used to treat and prevent malaria in China a few decades ago, but it then fell into disfavor because of increasing drug resistance.^10^ More recently, piperaquine has become a component of another ACT, dihydroartemisinin/piperaquine. The 8-aminoquinoline, primaquine, has some activity against erythrocytic parasites, but it is used principally to eliminate parasite liver stages, including the exoerythrocytic forms that precede erythrocytic infection in all species and the hypnozoites that cause latent infections with *P*. *vivax* and *Plasmodium ovale*. Primaquine also acts against gametocytes, thereby lowering transmission of parasites to mosquito vectors. Quinine is an arylamino alcohol that is the oldest antimalarial drug, used as cinchona bark since the 1600s and in its pure form since 1820.^11^ Quinine is quite hard to tolerate, and its use is best limited to the treatment of severe malaria. Important malaria-related drugs are mefloquine and lumefantrine, components of the ACTs artesunate/mefloquine and artemether/lumefantrine.

Antifolates target parasite dihydrofolate reductase (DHFR) and dihydropteroate synthase (DHPS). Sulfadoxine/pyrimethamine has the distinct advantage of single-dose therapy, but its treatment efficacy is seriously limited by drug resistance. The naphthoquinone atovaquone acts against the mitochondrial cytochrome bc_1_ complex. Combined with the DHFR inhibitor proguanil as Malarone, it offers effective albeit expensive therapy and chemoprophylaxis for falciparum malaria, although it is noteworthy that the synergy of this combination appears to be independent of inhibition of folate synthesis. Several antibiotics that are prokaryotic protein synthesis inhibitors have antimalarial activity because of action against the protein synthesis machinery of the apicoplast organelle.^12^ Doxycycline is used for chemoprophylaxis against malaria, and doxycycline or clindamycin are combined with quinine to treat falciparum malaria.

The most important new class of antimalarials is the artemisinins, which were developed from a natural product remedy in China.^13^ Artemisinin is a potent antimalarial, but the derivatives artesunate, artemether, and dihydroartemisinin are most widely used as components of ACT regimens. Indeed, the use of artemisinins outside combination regimens is strongly discouraged by the WHO because of fear of selecting for resistance to this important class of drugs. Artemisinins are highly effective against acute malaria, but short acting, so combination with longer-acting drugs in ACTs allows short (3-day) courses of treatment that protect against the selection of resistance to the artemisinin component.^7^ Because of its rapid action, intravenous artesunate is also the new gold standard for the treatment of severe falciparum malaria, with documented survival advantages compared with intravenous quinine.^14^,^15^

## Antimalarial Drug Resistance

The efficacies of many antimalarial drugs are limited by drug resistance, and recent evidence suggests that parasites are becoming resistant to the newest agents. However, the extent of resistance varies, such that in many cases drugs with resistance concerns are nonetheless offering good effectiveness for the treatment and control of malaria. Resistance has been described for nearly all available drugs, as is discussed below. For many drugs the extent of resistance is uncertain and mechanisms of resistance are unknown, and thus the opportunity to glean data from the 10 ICEMR surveillance sites is highly valuable. Resistance can be assessed by clinical trials comparing antimalarial efficacies of different agents, ex vivo/in vitro assessment of sensitivities of cultured *P*. *falciparum*, evaluation of genetic polymorphisms associated with resistance, or by assessing the selective pressure of antimalarial treatment on subsequent infections. Studies considering all of these factors have shed light on the extent of resistance and on mechanisms of resistance.

### Resistance mediated by transporter mutations.

The *P*. *falciparum* genome encodes multiple predicted transporters.^16^ Polymorphisms in transport proteins can mediate resistance to many agents active against cancer and infectious diseases via enhancing efflux of the drugs from cells.^17^ It appears that a number of plasmodial proteins transport different drugs and that polymorphisms in these proteins may impact on drug sensitivity.^18^

#### pfmdr1.

Polymorphisms in the *P*. *falciparum* multidrug resistance-1 (*pfmdr1*) gene, which encodes the P-glycoprotein homolog, impact on sensitivity to multiple antimalarial drugs.^19^–^21^ In humans, P-glycoprotein polymorphisms are associated with resistance to cancer drugs.^22^ In *P*. *falciparum*, the function of the *pfmdr1* product is unknown, but the protein localizes to the membrane of the food vacuole, the site of action of a number of drugs, suggesting that it is a drug transporter.^23^ Data on associations between *pfmdr1* polymorphisms and drug sensitivity are complex, but overall suggest that changes in *pfmdr1* sequence or copy number alter transport of multiple drugs in or out of the parasite food vacuole, with individual polymorphisms leading to opposite effects on different drugs.^24^ Increased copy number of *pfmdr1*, which is prevalent in southeast Asia, has been associated with extensive use of mefloquine.^25^ Experimental evidence indicates that *pfmdr1* amplification also leads to decreased sensitivity to quinine, lumefantrine, and artemisinin.^26^ Mutations at *pfmdr1* N86Y and D1246Y (for this and other *P. falciparum* genes, wild type sequence is based on the 3D7 reference strain), which are common in Africa, have been linked to decreased sensitivity to chloroquine and amodiaquine, but increased sensitivity to lumefantrine, mefloquine, and artemisinins.^27^–^31^ Other polymorphisms primarily seen outside Africa (including 1034C and 1042D) are associated with altered sensitivity to lumefantrine, mefloquine, and artemisinins.^26^,^29^,^32^–^34^ Considering infections that emerge soon after prior therapy, amodiaquine-containing regimens selected for the 86Y and 1246Y mutant alleles^35^–^37^ and for parasites with decreased in vitro sensitivity to the active metabolite monodesethylamodiaquine^38^ in subsequent infections. The selective pressure of the related aminoquinoline piperaquine seems less marked than that of amodiaquine, but prior use of the drug also selected for the *pfmdr1* 86Y and 1246Y mutations.^31^,^39^ In contrast, therapy with artemether/lumefantrine selected for the N86 and D1246 wild type alleles in subsequent infections within 60 days of prior therapy.^31^,^35^,^36^,^39^–^43^ Importantly, impacts of *pfmdr1* polymorphisms on drug sensitivity are modest, correlations between particular polymorphisms and treatment efficacy have not been seen, and the ACTs artesunate/amodiaquine and artemether/lumefantrine remain highly efficacious for the treatment of uncomplicated falciparum malaria in Africa.^44^,^45^ However, as seen for chloroquine and amodiaquine, *pfmdr1* polymorphisms may contribute, with additional polymorphisms, to resistance to increasingly used components of ACTs.

#### pfcrt.

Soon after the identification of *pfmdr1*, it became clear that polymorphisms in this gene are not the primary mediators of chloroquine resistance. Subsequently, analysis of progeny of a genetic cross between chloroquine sensitive and resistant strains led to the identification of *pfcrt*,^46^ which encodes a food vacuole membrane protein that is predicted to be a member of the drug/metabolite transporter superfamily.^47^,^48^ The function of *pfcrt* is unknown, but apparently essential, as disruption of the gene has not been possible.^49^ *pfcrt* is highly polymorphic, but one single nucleotide polymorphism (SNP), K76T, is the primary mediator of chloroquine resistance.^49^,^50^ The 76T mutation appears to act principally by increasing the export of chloroquine from the food vacuole, but the mechanism of *pfcrt* 76T-mediated chloroquine resistance is incompletely understood.^49^ Other *pfcrt* SNPs always accompany 76T in field isolates, and these likely encode compensatory mutations that allow parasites containing 76T to maintain adequate fitness; some other SNPs may also contribute directly to the drug resistant phenotype. The 76T mutation also mediates decreased sensitivity to monodesethylamodiaquine, and studies with genetically modified parasites have shown it to mediate increased susceptibility to mefloquine and artemisinins,^51^ suggesting the same reciprocal relationship between sensitivities to aminoquinolines and other drugs as described for certain *pfmdr1* polymorphisms.

#### Pfmrp1.

*Plasmodium falciparum* multidrug resistance protein-1 (Pfmrp1) is a member of the ABC transporter superfamily.^24^ In studies of culture-adapted *P. falciparum*, SNPs in *pfmrp1* were linked to decreased sensitivity to chloroquine and quinine.^16^ Two SNPs that appear to be common in African parasites, I876V and K1466R, were selected by prior treatment with artemether/lumefantrine^52^ and sulfadoxine/pyrimethamine,^53^ respectively, although these SNPs were not associated with altered drug sensitivity in African isolates.^31^ *pfmrp1* mutations appear to differ between continents; some SNPs in northeast Myanmar isolates were associated with reduced susceptibilities to chloroquine, mefloquine, pyronaridine, and lumefantrine.^54^ Disruption of the *pfmrp1* gene yielded parasites with diminished growth and increased sensitivity to chloroquine and other drugs, suggesting a role for this protein in the efflux of antimalarial drugs from the parasite and in parasite fitness.^55^

#### Sodium transporters.

Quantitative trait locus analysis identified three genes predicted to play roles in the responsiveness of *P. falciparum* to quinine, *pfcrt*, *pfmdr1*, and *pfnhe1*, which encode a putative sodium–hydrogen exchanger and are highly polymorphic.^56^ *pfatp4* encodes a *P. falciparum* plasma membrane protein that appears to be a sodium efflux pump.^57^ Recent studies have shown that three different classes of potent antimalarial compounds, spiroindolones, pyrazoleamides, and dihydroisoquinolones, all target *pfatp4*.^57^–^59^ Mutations in *pfatp4* have been linked to altered sensitivity to these candidate antimalarials.^58^–^60^ In addition, screening of the “Malaria Box” chemical library identified 28 compounds of diverse chemotypes that affected parasite Na^+^ and pH regulation in a manner consistent with PfATP4 inhibition.^61^ A recent clinical trial of the spiroindolone KAE609 demonstrated excellent efficacy against falciparum and vivax malaria.^62^

### Resistance to quinine.

Resistance to quinine, the oldest antimalarial drug, was reported first in Brazil^63^ and later in southeast Asia.^64^,^65^ Quinine resistance is associated with polymorphisms in several transporters. As stated earlier, SNPs in *pfmdr1*, *pfcrt*, and *pfmrp1* are linked to decreased sensitivity to quinine. In addition, *pfmdr1* gene amplification can also lead to quinine resistance.^66^ Recent studies evaluating associations between polymorphisms in a *pfnhe1* microsatellite, in vitro parasite sensitivity, and clinical responses to various drugs have been inconsistent, but these polymorphisms appear to have a modest impact on sensitivity of parasites to quinine, and possibly other drugs.^67^–^72^ In vitro allelic exchange to reduce the expression of *pfnhe1* by ~50% led to a 30% increase in quinine sensitivity in some, but not other parasite strains.^73^

### Resistance to antifolates.

The parasite-specific antimetabolite, pyrimethamine, is usually discussed in combination with its partner drug sulfadoxine (known as SP or Fansidar). Pyrimethamine was first used as an individual drug, but resistance was seen within a year in both *P. vivax* and *P. falciparum*.^74^,^75^ The combination of sulfa drugs and pyrimethamine proved to be potent in the laboratory, as well as in the field against chloroquine-resistant uncomplicated malaria but, again, resistance appeared rapidly in the Asia Pacific regions in the late 1970s, as well as in South America.^76^–^79^

Molecular genetic studies attribute pyrimethamine and sulfa resistance to mutations in the genes coding for the target enzymes DHFR and DHPS.^80^,^81^ These markers have been useful for tracking sulfadoxine/pyrimethamine resistance across the globe, and show particular promise with new multiplex strategies.^82^ In the 1990s, sulfadoxine/pyrimethamine found increasing use in Africa to treat widespread chloroquine-resistant malaria, before sulfadoxine/pyrimethamine resistance followed. Molecular epidemiology studies utilizing DNA microsatellite sequences flanking the *dhfr* gene point to transfer of pyrimethamine resistance from Asia to Africa, possibly from a single ancestor and possibly before sulfadoxine/pyrimethamine use even began in Africa.^83^ In contrast, resistance to sulfa partners through *dhps* mutations seemed to occur through de novo events both in sub-Saharan Africa and in Asia.^84^,^85^ Sulfadoxine/pyrimethamine is no longer recommended as a first-line drug for the treatment of *P. falciparum*. However, it continues to be used in ACT combinations in most parts of India,^86^ for intermittent preventive therapy in pregnant women in Africa,^87^ and for seasonal malaria chemoprevention in children in the sub-Sahel of Africa,^88^ although widespread resistance limits these interventions.^89^,^90^ Perhaps not surprisingly, sulfadoxine/pyrimethamine resistance in *P. vivax* appeared in Asia and the Pacific Islands, where *P. falciparum* and *P. vivax* coexist.^91^–^94^

### Resistance to artemisinin family drugs.

Since artemisinins play an indispensable role in current malaria therapies, artemisinin resistance has received wide recent attention. In the Cambodia–Thailand border region of southeast Asia, an epicenter of antimalarial drug resistance, declining efficacy of the artesunate/mefloquine combination was noted,^95^ and clinical resistance to artesunate, manifested as delayed clearance of parasitemia after therapy, but not generally as full-blown treatment failure, was documented in 2008.^96^–^98^ The delayed parasite clearance phenotype does not correspond to increased artemisinin half maximal inhibitory concentration (IC_50_) values, as determined by standard in vitro assays, but does correspond to decreased susceptibility assessed 72 hours after a pulse of dihydroartemisinin using the new ring-stage survival assay.^99^,^100^ Attempts to select resistance to artemisinins in vitro using constant or pulsed drug selection pressure initially identified *pfmdr1* amplification and increased antioxidant levels in selected parasites.^101^,^102^ In field-based studies, genome-wide association studies identified regions on chromosome 13 linked to delayed parasite clearance.^103^,^104^ Using a combined resistance selection and genomic approach, Ariey and others^105^ identified mutations in the propeller domain of the *P. falciparum* kelch (*K13*) gene (PF3D7_1343700) associated with delayed parasite clearance after artemisinin therapy in southeast Asia. Very recently, using clinical and molecular data, the extent of artemisinin resistance has been delineated, with delayed clearance and *K13* mutations common in parts of Cambodia, Thailand, Myanmar, and Vietnam, but not in other areas of Asia or Africa.^106^–^108^ Other reports from Cambodia have shown recrudescent infections after treatment with dihydroartemisinin/piperaquine, raising the concern that resistance to artemisinin partner drugs has been facilitated by the spread of artemisinin resistance.^109^ In African parasites, although *K13* gene polymorphisms are common, including some mutations in the propeller domain, the specific mutations associated with artemisinin resistance in southeast Asia have not been seen.^110^–^112^ Parasites with introduced *K13* mutations showed enhanced survival after a dihydroartemisinin pulse, confirming the role of these mutations in resistance.^113^ The transcriptomes of resistant parasites showed increased expression of unfolded protein response pathways and prolonged ring-stage development, offering insights into the mechanism of artemisinin resistance.^114^

### Resistance to Malarone.

Atovaquone is a potent inhibitor of electron transport, and studies identified the target of this drug as the critical quinone-binding sites of cytochrome b.^115^,^116^ When the drug is used alone, resistance develops rapidly and recrudescence after therapy is common. Resistance is conferred by single-point mutations in the cytochrome b (*Pfcytb*) gene. *Pfcytb* mutations 268S and 268N were associated with Malarone treatment failure.^117^,^118^ However, treatment failure has also been reported in the absence of these mutations.^119^–^121^

## ICEMR Data Cocncerning Antimalarial Drug Resistance

Data from ICEMR sites, collected both before and during enactment of the ICEMR programs, offer insight into global drug resistance trends. Research activities at different ICEMR sites entail clinical studies, ex vivo/in vitro assays, and molecular studies (Table 2 ).

### Clinical observations.

Clinical trials in west Africa, Uganda, south Asia, and Papua New Guinea (PNG) have generally shown excellent antimalarial efficacy for the ACTs artemether/lumefantrine, artesunate/amodiaquine, and dihydroartemisinin/piperaquine (Supplemental Table 1). In high transmission settings, new infections after ACT therapy may be common, but true recrudescences after treatment have been very uncommon. In India, particularly in the northeast along the Myanmar border, where artesunate/sulfadoxine–pyrimethamine combinations are being discontinued, there have been excellent responses to artesunate/mefloquine, artesunate/amodiaquine, and dihydroartemisinin/piperaquine (Supplemental Table 1). Even in low-endemicity areas at the China–Myanmar border, where artemisinins have the longest history of use, excellent efficacy of the ACTs for treatment of falciparum malaria has been seen.^122^ On the other hand, an effectiveness study in Uganda showed a failure rate of 31% after treatment with quinine.^123^ In regions of *P. falciparum*/*P. vivax* co-endemicity, *P. falciparum* typically shows rapid responses to control efforts, whereas *P. vivax* prevalence subsides more slowly. In northeast Myanmar, follow-ups of *P. vivax* cases after chloroquine/primaquine treatment indicated an increase in cases with recurrent parasitemia within 28 days compared with a prior report,^124^ suggesting the emergence of chloroquine resistance.^125^ Ongoing clinical efficacy studies at the ICEMR sentinel sites will be important to offer a longitudinal appreciation of drug efficacy and provide a scientific basis to guide local drug policy (Table 3 ).

### Ex vivo and in vitro studies.

Studies on ex vivo (parasites studied immediately after collection from infected patients) or in vitro (parasites studied after culture adaptation) antimalarial drug sensitivity of *P. falciparum* have been conducted by ICEMR groups from west Africa, Uganda, south Asia, southeast Asia, and PNG (Supplemental Table 2). Ex vivo studies have the advantage of testing samples directly from patients without potential selection biases due to constraints of culturing and cryopreservation. However, the results may be confounded by the presence of multiple clones of parasites that differ in sensitivities to the test drugs. In vitro assays performed with culture-adapted parasite clones allow assays to include multiple biological replications and provide better opportunities for subsequent genetic analysis. In general, these studies have shown that African parasites have varied sensitivities to chloroquine and amodiaquine, and good sensitivities to dihydroartemisinin, the active metabolite of all artemisinin derivatives, and to the ACT partner drugs lumefantrine, mefloquine, and piperaquine.^31^,^126^,^127^ In Uganda, increased deployment of artemether/lumefantrine was linked to some decrease in in vitro susceptibility to lumefantrine.^31^ In Thailand, reduced lumefantrine susceptibility might have resulted from extensive use of mefloquine, another aminoalcohol.^128^ Clinical and in vitro resistance to quinine has been seen in southeast Asia, but not consistently in Africa. In Senegal and Uganda, for example, *P. falciparum* parasites appeared to be susceptible to quinine in vitro.^126^,^129^ In comparison, data from southeast Asia showed a mean IC_50_ greater than 500 nM.^68^ Similar to African parasites, southeast Asian isolates were generally sensitive to artemisinins and the ACT partner drugs lumefantrine and mefloquine.^130^ Yet, longitudinal studies revealed gradual decrease of susceptibility to piperaquine, and a high correlation between chloroquine and piperaquine IC_50_ values.^131^ However, only limited results are available, and considerations of ex vivo/in vitro results is complicated by varied methodologies used by different groups, difficulties of interpreting results for polyclonal infections, and uncertain correlations between in vitro findings and clinical efficacy. Commonly used ex vivo/in vitro assays measure the parasite histidine-rich protein-2 by enzyme-linked immunosorbent assay, replication of parasite DNA by isotope incorporation, or use of a fluorescent dye such as SBYR Green I.^132^,^133^ To enhance comparisons among sites, ex vivo/in vitro assays should consider the inclusion of a standard laboratory strain (such as 3D7) as an internal control. Further, the new ring-stage survival assay^99^ should be adopted in multiple ICEMR sites to monitor the emergence and spread of artemisinin resistance. Some ICEMRs also have prevalent transmission of vivax malaria. Ex vivo drug assays for *P. vivax* are also being conducted (Table 3), but the assays are constrained by difficulties of *P. vivax* culture and the appreciation that assays for certain drugs (e.g., chloroquine) require a high proportion of parasites at the ring stage and a high parasitemia.

### Genotyping drug resistance–mediating polymorphisms.

Studies of genetic polymorphisms associated with drug resistance are technically much simpler than in vitro studies of parasite sensitivity, and so results are more widely available. Studies of *P. falciparum* genetic polymorphisms have been conducted by ICEMR groups from west Africa, southern Africa, Uganda, south Asia, southeast Asia, and PNG (Supplemental Tables 3 and 4). As has already been well documented in past studies, the prevalence of a number of polymorphisms that impact on drug sensitivity varies greatly around the world. Also of interest are changes in polymorphism prevalence over time. In Uganda, parasites have demonstrated marked changes in the prevalence of some key polymorphisms over the last decade, coincident with changes in treatment practices for malaria from chloroquine to chloroquine/sulfadoxine–pyrimethamine to artemether/lumefantrine. Most notably, the prevalence of three wild type alleles, *pfcrt* K76, *pfmdr1* N86, and *pfmdr1* D1246, has all increased markedly in recent years^134^ and this increase was greater in children treated with artemether/lumefantrine for all episodes of malaria than in those treated with dihydroartemisinin/piperaquine.^39^ This is in sharp contrast to the *P. falciparum* parasites at the China–Myanmar border area, where *pfcrt* 76T and 220S remained almost fixed in a recent study.^135^ Recently, the Uganda ICEMR group showed that therapy with artemether/lumefantrine selects for the wild-type polymorphisms associated with decreased lumefantrine efficacy and, in ex vivo studies, for parasites with diminished lumefantrine sensitivity.^31^ Importantly, despite these changes, sensitivity to lumefantrine remains quite good, and artemether/lumefantrine treatment efficacy is excellent. However, recent unpublished trials (Yeka and others, unpublished data) showed that in 2013–2014 artemether/lumefantrine was less efficacious than artesunate/amodiaquine at three different sites in Uganda, a change in relative treatment efficacy compared with older findings.^44^,^136^ These results suggest that recent changes in treatment practices have led to changes in *P. falciparum* in Uganda that have mediated decreased antimalarial efficacy of artemether/lumefantrine, the first-line antimalarial drug in the country. An urgent issue that the ICEMRs are addressing is monitoring of the emergence and/or spread of artemisinin resistance.^110^ The identification of the *K13* gene as a molecular marker for artemisinin resistance will facilitate large-scale surveillance.^137^

## Resistance and Fitness

One of the challenges to studying the interplay between parasite drug resistance and fitness is the lack of a direct measure for fitness. Comparison of relative growth rates in vitro or ex vivo is the commonly used approach, although growth rates may not represent relative fitness in the natural host. Assessment of parasite survival in the field provides an improved measure, although analyses are challenging. It has long been observed that *P. falciparum* genetic mutations that confer drug resistance are associated with altered biological fitness of the parasite.^138^–^140^ However, various investigators have reported both increased^141^–^143^ and decreased^144^–^146^ fitness in resistant parasites. The latter would seem intuitive from initial low prevalence of innate resistance observed in the field for some antimalarials such as mefloquine and atovaquone, presumably owing to the mutant parasites being outcompeted by the wild type before start of drug use.^117^,^147^,^148^ More compelling evidence for a fitness cost of resistance has been documented by reemergence of sensitive parasites, virtually replacing highly prevalent resistant strains, following withdrawal of drug (chloroquine or sulfadoxine–pyrimethamine) pressure in the population.^149^–^154^ The rub is that in other areas under similar conditions, reemergence has occurred much more slowly or not at all.^155^–^159^ Similarly cogent evidence of a fitness cost to resistance has been demonstrated by decreased prevalence of resistant parasites after the dry season in west Africa, when there is relatively little drug selection pressure.^160^–^162^ Again, other studies have had contrasting results.^163^,^164^

Global data from the ICEMRs afford an opportunity for a concerted evidence base on associations between resistance and fitness, and potentially on the de facto risk factors for the emergence or suppression of drug resistance in the field. So far drug selection pressure, herd immunity,^153^,^160^ and ecological differences^146^,^165^,^166^ have been shown to impinge on relative fitness of drug-resistant and drug-sensitive parasites in the wild. Although combination of antimalarial compounds with opposing resistance mechanisms have been used to suppress the emergence of drug resistance in laboratory isolates,^167^ opposite resistance selection has also been observed in the field between 4-aminoquinolines (chloroquine, amodiaquine) and artemisinins.^27^,^168^–^170^ Data from Uganda showed significantly lower prevalence of symptoms among children infected with parasites containing chloroquine resistance mutations compared with those infected with wild-type parasites, consistent with greater virulence for wild-type parasites.^171^ Field data from the southern Africa ICEMR suggest a role for the vector in selecting drug resistance polymorphisms, with significant differences in prevalence of SNPs that mediate resistance to aminoquinolines and antifolates between parasites infecting mosquitoes and people.^172^,^173^ So far, *Anopheles arabiensis*^172^,^173^ and more recently *Anopheles funestus* (Matsena and others, unpublished data) have both been shown to exert selection on drug resistance polymorphisms. This would seem to explain the role of ecology in governing resistance,^165^,^166^ which can differ between different regions of the same country. The unexpected link between vector control and prevalence of drug-resistant malaria parasites in some areas^174^–^177^ but not others^178^ also seems consistent with vector selection. More detailed data from multiple ICEMRs will be instrumental in improving our understanding of the interplay between drug resistance and fitness, and hopefully the development of more effective strategies for the containment of drug-resistant malaria.

## Conclusion and Future Studies

In an evolutionary arms race between malaria parasites and a series of therapeutic interventions, the parasites have consistently been able to develop resistance to each new class of drugs. The emergence of parasites resistant to artemisinins in southeast Asia and altered sensitivities to artemisinin partner drugs pose great threats to efforts to control and, eventually, eradicate malaria. Specifically, previous failures of the ACTs artesunate/mefloquine and artesunate/amodiaquine have recently been followed by frequent failures of dihydroartemisinin/piperaquine in parts of Cambodia, and decreasing sensitivity to lumefantrine may further threaten artemether/lumefantrine. It is thus of high priority to continue surveillance of ACT efficacy, the ex vivo and in vitro activities of ACT components, and molecular markers that may mediate resistance to these drugs. For artemisinins, mutations in the *K13* gene offer markers for the delayed parasite clearance phenotype that is now common in parts of southeast Asia. Mutations in the putative drug transporters *pfmdr1* and *pfcrt* mediate altered sensitivity to multiple artemisinin partner drugs, including amodiaquine, mefloquine, lumefantrine, and piperaquine, although different drugs are impacted in opposite directions. Additional parasite polymorphisms are likely important in drug responsiveness, and an improved understanding of the roles of these polymorphisms is an important goal. Additional studies on the influence of drug resistance on parasite fitness may enable the identification of optimal dosing strategies, including, possibly, rotating of regimens. Further, understanding how mosquitoes mediate the spread of drug resistance and use of evolution-proof mosquito control measures may help to deter resistance spread, enabling the regional elimination and eventual eradication of malaria.

## Supplementary Material

Supplemental Datas.

## Figures and Tables

**Table 1:** Currently used antimalarial drugs

Class	Drug	Use
4-Aminoquinoline	Chloroquine	Treatment of non-falciparum malaria
	Amodiaquine	Partner drug for ACT
	Piperaquine	ACT partner drug with dihydroartemisinin as ACT
8-Aminoquinoline	Primaquine	Radical cure and terminal prophylaxis of *Plasmodium vivax* and *Plasmodium ovale*; gametocytocidal drug for *Plasmodium falciparum*
		Radical cure of *P. vivax* and *P. ovale*
	Quinine	Treatment of *P. falciparum* and severe malaria
Arylamino alcohol	Mefloquine	Prophylaxis and partner drug for ACT for treatment of falciparum
	Lumefantrine	Combination with artemether as ACT
Sesquiterpene lactone endoperoxides	Artemether	ACT: combination with lumefantrine
	Artesunate	ACT; treatment of severe malaria
	Dihydroartemisinin	ACT: combination with piperaquine
Mannich base	Pyronaridine	Combination with artesunate as ACT
Antifolate	Pyrimethamine/sulfadoxine	Treatment of some chloroquine-resistant parasites; Combination with artesunate as ACT
Naphthoquinone/antifolate	Atovaquone/proguanil	Combination for prophylaxis and treatment of *P. falciparum* (Malarone)
Antibiotic	Doxycycline	Chemoprophylaxis; treatment of *P. falciparum*
	Clindamycin	

ACT = artemisinin-based combination therapy.

**Table 2:** Drug resistance surveillance activities in ICEMRs and ICEMR regions

ICEMR	Drug efficacy trials	Parasite clearance data	Ex vivo drug sensitivity*	In vitro drug sensitivity†	Drug resistance polymorphisms
West Africa	Yes	Yes	Yes	Yes	Yes
Southern Africa	No	Yes	No	No	Yes
Malawi	Yes	Yes	No	No	Yes
Uganda	Yes	Yes	Yes	Yes	Yes
South Asia	Yes‡	Yes	No	Yes	Yes
Southeast Asia	Yes	Yes	No	Yes	Yes
PNG	Yes	No	Yes	No	No

ICEMR = the International Centers of Excellence for Malaria Research; PNG = Papua New Guinea.

* Characterization of sensitivity in fresh samples from infected subjects.

† Characterization of sensitivity in culture-adapted parasites.

‡ Conducted by ICEMR partners.

**Table 3:** Studies of *Plasmodium vivax*

ICEMR/region	Drug efficacy trials		Ex vivo drug sensitivity	Drug resistance polymorphisms	
	ACT	CQ/primaquine		*pvmdr1*	*pvdhfr*
Southeast Asia	No	Yes	Yes	Yes	No
South Asia	Yes	Yes	Yes	Yes	Yes
PNG	AL, DP, ART/NQ, ART-SP	CQ-SP and AQ-SP, no primaquine	No	Yes	Yes

ACT = artemisinin-based combination therapy; AL = artemether/lumefantrine; ART = artemisinin; AQ = amodiaquine; CQ = chloroquine; DP = dihydroartemisinin/piperaquine; ICEMR = the International Centers of Excellence for Malaria Research; MQ = mefloquine; NQ = naphthoquine; PNG = Papua New Guinea; SP = sulfadoxine/pyrimethamine.
