# Supplementary material for: Antimalarial Drug Resistance: Literature Review and Activities and Findings of the ICEMR Network
Source: Am J Trop Med Hyg. 2015 Sep 2;93(3 Suppl):57–68. doi: 10.4269/ajtmh.15-0007 (PMC4574275; doi:10.4269/ajtmh.15-0007)
Supplement: Supplementary file 1 [file SD1.pdf]

Supplemental Table 1

Drug efficacy trials including ACT regimens from the ICEMR countries/regions

ICEMR	Specific location	Dates of trial	Trial comparison	Participants (*N*)	Risk failure 28 days, uncorrected	Risk failure 28 days, corrected	Ref.
West Africa	Thies	2011–2013	AL	147	2/147 (1%)	(1%)	–
	Dioro	2011–2013	AL	37	12/37 (32%)	(0%)	–
	Gambissara	2011–2013	AL	25	1/25 (4%)	(0%)	–
Uganda	Kampala	2002–2003	CQ/SP vs. AQ/SP vs. AS/AQ	384	CQ/SP 56%; AQ/SP 17%; AS/AQ 17%	CQ/SP 41%; AQ/SP 10%; AS/AQ 4%	1
	Apac, Arua, Jinja, Tororo	2002–2004	CQ/SP vs. AQ/SP vs. AS/AQ	2,160	CQ/SP 63–88%; AQ/SP 28–59%; AS/AQ 19–74%	CQ/SP 22–46%; AQ/SP 7–18%; AS/AQ 4–12%	2
	Tororo	2004–2005	AS/AQ vs. AL	403	AS/AQ 66%; AL 51%	AS/AQ 0%; AL 1.0%	3
	Kampala	2004–2006	AQ/SP vs. AS/AQ vs. AL	~200/arm	AQ/SP 26.1%; AS/AQ 17.4%; AL 6.7%	AQ/SP 14.1%; AS/AQ 4.6%; AL 1.0%	4
	Apac	2006	AL vs. DP	417	AL 29%; DP 11%	AL 8.9%; DP 1.9%	5
	Kanungu	2006	AL vs. DP	408	AL 17.3%; DP 3.8%	AL 3.2%; DP 0.9%	6
	Tororo	2007–2008	AL vs. DP	671	AL 35%; DP 11%	AL 1.0%; AS/AQ 0.3%	7
	Kampala	2007–2008	QN vs. AL	178	QN 35.3%; AL 4.1%	QN 23.1%; AL 4.1%	8
	Kampala	2004–2008	AQ/SP vs. AS/AQ vs. AL	1,464	AQ/SP 26.7%; AS/AQ 16.1%; AL 6.1%	AQ/SP 16.0%; AS/AQ 3.5%; AL 0.8%	9
	Tororo	2007–2012	AL vs. DP	5,564	AL 51.0%; DP 8.9%	AL 3.7%; DP 4.4% (63 days)	10
	Apac	2013	AL vs. AS/AQ	100/arm	AL 32.0%; AS/AQ 15.0%	AL 2.1%; AS/AQ 0%	–
	Mubende	2013	AL vs. AS/AQ	100/arm	AL 54.9%; AS/AQ 35.0%	AL 4.3%; AS/AQ 0%	–
	Kanungu	2014	AL vs. AS/AQ	100/arm	AL 50.0%; AS/AQ 38.0%	AL 41.1%; AS/AQ 0%	–
South Asia	Jharkhand, Odiisha	2007–2008	AS/AQ vs. AQ	300	AS/AQ 22%; AQ 13%	AS/AQ 5–10%; AQ 5%	11
	Assam, Goa, Karnataka	2006–2007	DP vs. AS/MQ	150	DP 10% vs. AS/MQ 18%	DP 1% vs. AS/MQ 0%	12
	Goa, Karnataka	2007–2008	AS/MQ	77	AS/MQ 1%	AS/MQ 0%	13
	Arunachal Pradesh, Tripura, Mizoram	2012	AS/SP	169	AS/SP 33%	AS/SP 20%	14
	Nine sites	2009–2010	AS/SP vs. AS/SP//PQ	1,335	–	–	15
	Seven countries	2007–2008	AS/PY vs. AS/MQ	1,271	AS/PY 4%; AS/MQ 5%	–	16
PNG	Alexishaven, Kunjinini	2006–2008	AL vs. DP vs. CQ/SP vs. ART/SP	Pf: 110–127/arm; Pv: 39–61	Pf: AL 7%, DP 14%, CQ/SP 32%, ART/SP 13%; Pv: AL 51%, DP 16%, CQ/SP 49%, ART/SP 49%	Pf: AL 3%, DP 10%, CQ/SP 15%, ART/SP 10%	17
	Mugil	2011–2013	AL vs. ART/NQ	Pf:100/arm; Pv: 20 and 27	Pf: AL 2%, ART/NQ 0%; Pv: AL 35%, ART/NQ 0%	Pf: AL 1%, ARCO 0%; Pv: AL 20%, ARCO 0%	–

ACT = artemisinin-based combination therapy; AL = artemether/lumefantrine; AQ = amodiaquine; ARCO = ART/NQ; ART = artemisinin; AS = artesunate; CQ = chloroquine; DP = dihydroartemisinin/piperaquine; ICEMR = the International Centers of Excellence for Malaria Research; MQ = mefloquine; NQ = naphthoquine, PNG = Papua New Guinea; PQ = piperaquine; PY = pyronaridine; QN = quinine; Ref. = references include both published data (shown below) and unpublished data; SP = sulfadoxine/pyrimethamine.

Supplemental Table 2

Ex vivo/in vitro drug efficacy results from the ICEMR countries/regions

ICEMR	Specific location	Date of collection	Source of samples	*N*	Assay	Mean (or median) IC_{50} (nM)								Ref.
						CQ	DEAQ	QN	LM	MQ	PQ	DHA	Other	
West Africa	Thies	2008	Adult, child	~400	Ex vivo; DAPI assay	30.7	9.6	NT	NT	34.5	NT	NT	–	18
		2009												
		2010				15.0	6.5	176.9	NT	44.6	NT	NT		
		2011				22.4	11.2	45.65	NT	32.6	NT	4.3		
		2012				76.1	14.5	239.9	119	42.1	101	2.1		
						36.5	14,3	178.2	168.1	36.6	173	1.9		
						23.8	6.65	182.4	316.9	38.5	20.3	2.0		
Uganda	Kampala	2006–2008	Child cohort	241	Ex vivo; HRP ELISA	101.1	66.4	94.4	0.51	–	6.1	0.55	–	19
	Tororo	2010–2012		286	Ex vivo; HRP ELISA	486.2	83.3	126.4	2.7	–	20.3	1.7	–	20
South Asia	Assam	2007	Child cohort	115	Ex vivo; microscopy	"221 uM/L"	–	–	–	–	–	–	–	21
	West Bengal	2008–2009	All ages	126	In vitro; hypoxanthine	54	–	–	–	–	–	–	–	22
Southeast Asia	China–Myanmar border	2006–2009	PCD samples	60	In vitro, SYBR Green	–	–	> 500	–	–	–	–	–	23
		2007–2009	PCD samples	51	In vitro, SYBR Green	–	–	–	5.9	50.4	–	23.0	AS 5.8	24
		2007–2010	PCD samples	63	In vitro, SYBR Green	252.7	–	–	–	–	28.4	–	–	25
PNG	Madang	2006–2007	Community survey	64	Ex vivo; pLDH ELISA	167	19.3	–	5.4	11.7	11.7	2.1	NQ 7.0	26

AS = artesunate; CQ = chloroquine; DAPI = 4′,6-diamidino-2-phenylindole; DEAQ = desethylamodiaquine; DHA = dihydroartemisinin; ELISA = enzyme-linked immunosorbent assay; HRP = horseradish peroxidase; ICEMR = the International Centers of Excellence for Malaria Research; LM = lumefantrine; MQ = mefloquine; NQ = naphthoquinone; NT = not tested; PCD = passive case detection; pLDH = *Plasmodium* lactate dehydrogenase; PNG = Papua New Guinea; PQ = piperaquine; QN = quinine; Ref. = references include both published data (shown below) and unpublished data.

SUPPLEMENTAL TABLE 3

Assessment of drug resistance polymorphisms in samples from the ICEMR countries/regions, transporters

ICEMR	Specific location	Date of collection	Source of samples	*N*	Single nucleotide polymorphism prevalence (%; WT/mixed/mutant)						Ref.
					Pfcrt K76T	*Pfmdr1* N86Y	*Pfmdr1* Y184F	*Pfmdr1* S1034C	*Pfmdr1* N1042D	*Pfmdr1* D1246Y	
West Africa	Pikine, Thies	2008	–	~700	44/3/53	88/2/10	64/4/32	100/0/0	75/1/24	100/0/0	18,27
		2009			49/5/46	81/5/14	42/7/51	100/0/0	82/0/8	100/0/0	
		2010			53/6/41	84/0/16	30/9/61	100/0/0	90/0/10	90/0/10	
		2011			54/3/43	89/11/0	41/9/50	Not done	89/0/11	Not done	
Zambia	Choma	2006	All ages, DBS	59	3.4/10.2/86.4	–	–	–	–	–	–
		2008	–	42	23.7/5.3/71.1	–	–	–	–	–	
		2009	–	30	37.9/0/62.1	–	–	–	–	–	
		2010	–	70	80.0/1.4/18.6	–	–	–	–	–	
Zimbabwe	Mutasa	2003	All ages, DBS	49	33.0/3.0/63.6	–	–	–	–	–	
		2013	–	54	97.0/0.0/3.0	–	–	–	–	–	
Uganda	Six sites	2002–2004	–	2,049	2.4/15.0/82.6	–	–	–	–	–	28
	Tororo	2004–2005	DBS	201	–	8/30/62	90/8/2	–	–	14/48/38	29
		2003–2004	–	79	0/1/99	10/71/19	75/23/2	–	–	2/48/50	30
		2005		201	0/0/100	9/38/53	85/14/1			17/40/43	
		2007		39	0/0/100	32/19/49	68/19/13			23/31/46	
		2008		147	0/2/98	23/61/16	71/15/14			24/34/42	
		2009		96	4/2/94	23/27/44	83/7/10			17/23/60	
		2010		111	0/4/96	44/37/19	37/41/22			19/50/31	
		2011		111	0/8/92	47/37/16	59/7/34			20/48/32	
		2012		79	17/16/67	51/41/8	68/6/26			53/34/13	
South Asia	Assam	2006–2008	All ages, PHC	200	0/1/99	30/-/68	–	–	–	–	31
				65	15/-/50	15/-/50	–	–	–	–	21
	Arunachal Pradesh	2006–2008		50	0/-/50	0/-/50	–	–	–	–	21
	West Bengal	2008–2009	All ages	126	45/-/55	39/-/61	100/-/0	34/-/66	100/-/0	54/0/46	22
	Madhya Pradesh	2004–2006	All ages, PHC	104	5/-/95	19/-/81	35/0/65	–	–	–	32
	Car Nicobar Islands	2008	Community	48	0/3/97	–	–	–	–	–	33
				204							
Southeast Asia	China–Myanmar border	2007–2009	PHC	51	–	2	27.5	–	7.8	–	24
		2007–2010	PHC	63	100	1.6	25.4	–	6.3	–	25

DBS = dry blood spots; ICEMR = the International Centers of Excellence for Malaria Research; PHC = public health center; Ref. = references include both published data (shown below) and unpublished data; WT = wild type.

Supplemental Table 4

Assessment of drug resistance polymorphisms in samples from the ICEMR countries/regions, antifolates

				Single nucleotide polymorphism prevalence (%; WT/mixed/mutant)										
				Pfdhfr					*Pfdhps*					
ICEMR/region	Specific location	Date of collection	*N*	A16V	N51I	C59R	S108N (*T)	I164L	S436A	A437G	K540E	A581G	A613T/S	Ref.
West Africa	Pikine, Thies	2003	700	–	47/13/40	46/13/41	26/7/67	100/0/0	77/14/9	26/6/67	100/0/0	100/0/0	100/0/0	18,27,34
		2008	–	–	8/4/88	7.8/3.2/89	3/2/95	100/0/0	74/3/23	79/1/20	100/0/0	100/0/0	100/0/0	
		2009	–	–	7/6/87	7/6/87	23/1/76	100/0/0	74/3/23	70/1/29	100/0/0	100/0/0	100/0/0	
		2010	–	–	6/6/88	6/6/88	9/3/88	100/0/0	86/1/13	36/1/63	100/0/0	100/0/0	100/0/0	
		2011	–	–	0/7/93	0/3/97	0/4/96	100/0/0	97/1/2	36/7/57	100/0/0	100/0/0	100/0/0	
		2012	–	–	2/15/83	2/16/82	1/95.8/3.2	100/0/0	90/1/9	36/5/54	100/0/0	100/0/0	100/0/0	
Zambia	–	2000	49	100//0	–	44.9/0.0/55.1	49.0/0.0/51.2	100/0/0	93.9/0/6.1	60.6/0.0/39.4	93.5/0.0/6.5	100/0/0	100/0/0	35,36
		2003	25	100/0/0	32.0/0.0/68.0	44.0/0.0/56.0	16.0/0.0/84.0	100/0/0	92/0/8	64.7/0.0/35.3	88.0/0.0/12.0	100/0/0	100/0/0	
		2006	136	1.5/0/98.5	20.8/4.8/74.4	40.3/8.5/51.2	5.9/8.1(1.5)/ 80.9(3.8)	100/0/0	92.9/0/7.1	78.6/0.0/21.4	83.9/0.0/16.1	100/0/0	100/0/0	
Malawi	Southern region	2012	3,000	–	0.77/0.64/98.6	1.19/3.14/95.0	0.28/2.02/97.7	99.66/0.34/0	99.4/0.4/0.2	1.09/1.66/97.24	2.38/1.19/96.44	95.7/2.16/2.08	91.3/8.7/0	–
Uganda	Kampala	1998–2003	812	–	8/4/88	34/20/56	2/12/88	–	–	24/22/64	20/20/60	–	–	37
	Six sites	2002–2004	2,049	–	0.4/3.1/96.5	19.8/24.3/55.8	0/0.4/99.6	99.8/0.2/0	–	9.2/14.2/76.6	10.8/19.0/70.2	–	–	28
	Tororo	2003–2004	79	–	0/6/94	7/37/56	0/0/100	80/0/0	–	2/14/84	2/26/72	100/0/0	–	30
		2005	201		1/2/97	7/31/62	1/2/97	99/1/0		2/12/86	1/13/86	98/1/1		
		2007	39		0/0/100	8/3/89	0/59/41	100/0/0		0/0/100	0/0/100	100/0/0		
		2008	147		1/0/99	3/6/91	0/23/77	99/1/0		1/1/98	0/4/96	88/11/1		
		2009	96		1/0/99	7/3/90	0/11/89	91/8/1		1/1/98	0/4/96	89/10/1		
		2010	111		0/1/99	9/11/80	1/16/83	88/11/1		1/2/97	1/4/95	87/4/9		
		2011	111		0/0/100	4/13/83	0/9/91	100/0/0		1/1/98	1/13/86	88/8/4		
		2012	79		0/0/100	8/6/86	0/2/98	100/0/0		0/5/95	0/4/96	98/2/0		
South Asia	Uttar Pradesh	2003–2004	35	100/0/0	97/0/3	24/0/66	3/0/97	97/0/3	100/0/0	91/0/9	100/0/0	100/0/0	100/0/0	38
	Assam	2003–2004	37	100/0/0	86/0/14	8/0/92	8/0/92	92/0/08	78/0/22	68/0/32	78/0/22	95/0/5	100/0/0	38
	Assam	2006–2008	200	99/0/1	80/0/20	3/0/97	0/0/100	86/0/14	75/025	40/0/60	61/0/39	79/0/21	76/0/22	31
	Madhya Pradesh	2004–2006	104	–	–	22/-/78	12/0/88	–	–	−88/-/12	96/-/4	97/0/3	–	32
	Goa	2012–2013	50	29/-/71	–	–	19/-/81	–	100/-/0	78/0/22	100/-/0	100/-/0	100/-/0	–
	Car Nicobar Island	2003–2004	33	100/0/0	10/0/90	0/0/100	0/0/100	12/0/88	54/0/46	20/0/80	80/0/20	80/0/20	100/0/00	39
Southeast Asia	China–Myanmar border	2010	117	0	63.2	99.1	99.1	78.6	62.3	98.3	87.2	37.6	0	40

ICEMR = the International Centers of Excellence for Malaria Research; PHC = public health center; Ref. = references include both published data (shown below) and unpublished data; WT = wild type.

SUPPLEMENTAL REFERENCES

1. Staedke SG, Mpimbaza A, Kamya MR, Nzarubara BK, Dorsey G, Rosenthal PJ, 2004. Combination treatments for uncomplicated falciparum malaria in Kampala, Uganda: randomised clinical trial. *Lancet 364:* 1950–1957.
2. Yeka A, Banek K, Bakyaita N, Staedke SG, Kamya MR, Talisuna A, Kironde F, Nsobya SL, Kilian A, Slater M, Reingold A, Rosenthal PJ, Wabwire-Mangen F, Dorsey G, 2005. Artemisinin versus nonartemisinin combination therapy for uncomplicated malaria: randomized clinical trials from four sites in Uganda. *PLoS Med 2:* e190.
3. Bukirwa H, Yeka A, Kamya MR, Talisuna A, Banek K, Bakyaita N, Rwakimari JB, Rosenthal PJ, Wabwire-Mangen F, Dorsey G, Staedke SG, 2006. Artemisinin combination therapies for treatment of uncomplicated malaria in Uganda. *PLoS Clin Trials 1:* e7.
4. Dorsey G, Staedke S, Clark TD, Njama-Meya D, Nzarubara B, Maiteki-Sebuguzi C, Dokomajilar C, Kamya MR, Rosenthal PJ, 2007. Combination therapy for uncomplicated falciparum malaria in Ugandan children: a randomized trial. *JAMA 297:* 2210–2219.
5. Kamya MR, Yeka A, Bukirwa H, Lugemwa M, Rwakimari JB, Staedke SG, Talisuna AO, Greenhouse B, Nosten F, Rosenthal PJ, Wabwire-Mangen F, Dorsey G, 2007. Artemether-lumefantrine versus dihydroartemisinin-piperaquine for treatment of malaria: a randomized trial. *PLoS Clin Trials 2:* e20.
6. Yeka A, Dorsey G, Kamya MR, Talisuna A, Lugemwa M, Rwakimari JB, Staedke SG, Rosenthal PJ, Wabwire-Mangen F, Bukirwa H, 2008. Artemether-lumefantrine versus dihydroartemisinin-piperaquine for treating uncomplicated malaria: a randomized trial to guide policy in Uganda. *PLoS One 3:* e2390.
7. Arinaitwe E, Sandison TG, Wanzira H, Kakuru A, Homsy J, Kalamya J, Kamya MR, Vora N, Greenhouse B, Rosenthal PJ, Tappero J, Dorsey G, 2009. Artemether-lumefantrine versus dihydroartemisinin-piperaquine for falciparum malaria: a longitudinal, randomized trial in young Ugandan children. *Clin Infect Dis 49:* 1629–1637.
8. Achan J, Tibenderana JK, Kyabayinze D, Wabwire Mangen F, Kamya MR, Dorsey G, D'Alessandro U, Rosenthal PJ, Talisuna AO, 2009. Effectiveness of quinine versus artemether-lumefantrine for treating uncomplicated falciparum malaria in Ugandan children: randomised trial. *BMJ 339:* b2763.
9. Clark TD, Njama-Meya D, Nzarubara B, Maiteki-Sebuguzi C, Greenhouse B, Staedke SG, Kamya MR, Dorsey G, Rosenthal PJ, 2010. Incidence of malaria and efficacy of combination antimalarial therapies over 4 years in an urban cohort of Ugandan children. *PLoS One 5:* e11759.
10. Wanzira H, Kakuru A, Arinaitwe E, Bigira V, Muhindo MK, Conrad M, Rosenthal PJ, Kamya MR, Tappero JW, Dorsey G, 2014. Longitudinal outcomes in a cohort of Ugandan children randomized to artemether-lumefantrine versus dihydroartemisinin-piperaquine for the treatment of malaria. *Clin Infect Dis 59:* 509–516.
11. Anvikar AR, Sharma B, Shahi BH, Tyagi PK, Bose TK, Sharma SK, Srivastava P, Srivastava B, Kiechel JR, Dash AP, Valecha N, 2012. Artesunate-amodiaquine fixed dose combination for the treatment of *Plasmodium falciparum* malaria in India. *Malar J 11:* 97.
12. Gargano N, Ubben D, Tommasini S, Bacchieri A, Corsi M, Bhattacharyya PC, Rao BH, Dubashi N, Dev V, Ghosh SK, Kumar A, Srivastava B, Valecha N, 2012. Therapeutic efficacy and safety of dihydroartemisinin-piperaquine versus artesunate-mefloquine in uncomplicated *Plasmodium falciparum* malaria in India. *Malar J 11:* 233.
13. Valecha N, Srivastava B, Dubhashi NG, Rao BH, Kumar A, Ghosh SK, Singh JP, Kiechel JR, Sharma B, Jullien V, Dash AP, Taylor WR, Anvikar AR, 2013. Safety, efficacy and population pharmacokinetics of fixed-dose combination of artesunate-mefloquine in the treatment of acute uncomplicated *Plasmodium falciparum* malaria in India. *J Vector Borne Dis 50:* 258–264.
14. Mishra N, Kaitholia K, Srivastava B, Shah NK, Narayan JP, Dev V, Phookan S, Anvikar AR, Rana R, Bharti RS, Sonal GS, Dhariwal AC, Valecha N, 2014. Declining efficacy of artesunate plus sulphadoxine-pyrimethamine in northeastern India. *Malar J 13:* 284.
15. Shah NK, Schapira A, Juliano JJ, Srivastava B, Macdonald PD, Poole C, Anvikar A, Meshnick SR, Valecha N, Mishra N, 2013. Nonrandomized controlled trial of artesunate plus sulfadoxine-pyrimethamine with or without primaquine for preventing posttreatment circulation of *Plasmodium falciparum* gametocytes. *Antimicrob Agents Chemother 57:* 2948–2954.
16. Rueangweerayut R, Phyo AP, Uthaisin C, Poravuth Y, Binh TQ, Tinto H, Penali LK, Valecha N, Tien NT, Abdulla S, Borghini-Fuhrer I, Duparc S, Shin CS, Fleckenstein L, 2012. Pyronaridine-artesunate versus mefloquine plus artesunate for malaria. *N Engl J Med 366:* 1298–1309.
17. Karunajeewa HA, Mueller I, Senn M, Lin E, Law I, Gomorrai PS, Oa O, Griffin S, Kotab K, Suano P, Tarongka N, Ura A, Lautu D, Page-Sharp M, Wong R, Salman S, Siba P, Ilett KF, Davis TM, 2008. A trial of combination antimalarial therapies in children from Papua New Guinea. *N Engl J Med 359:* 2545–2557.
18. Van Tyne D, Dieye B, Valim C, Daniels RF, Sene PD, Lukens AK, Ndiaye M, Bei AK, Ndiaye YD, Hamilton EJ, Ndir O, Mboup S, Volkman SK, Wirth DF, Ndiaye D, 2013. Changes in drug sensitivity and anti-malarial drug resistance mutations over time among *Plasmodium falciparum* parasites in Senegal. *Malar J 12:* 441.
19. Nsobya SL, Kiggundu M, Nanyunja S, Joloba M, Greenhouse B, Rosenthal PJ, 2010. In vitro sensitivities of *Plasmodium falciparum* to different antimalarial drugs in Uganda. *Antimicrob Agents Chemother 54:* 1200–1206.
20. Ochong E, Tumwebaze PK, Byaruhanga O, Greenhouse B, Rosenthal PJ, 2013. Fitness consequences of *Plasmodium falciparum pfmdr1* polymorphisms inferred from *ex vivo* culture of Ugandan parasites. *Antimicrob Agents Chemother 57:* 4245–4251.
21. Shrivastava SK, Gupta RK, Mahanta J, Dubey ML, 2014. Correlation of molecular markers, *Pfmdr1-N86Y* and *Pfcrt-K76T*, with in vitro chloroquine resistant *Plasmodium falciparum*, isolated in the malaria endemic states of Assam and Arunachal Pradesh, northeast India. *PLoS One 9:* e103848.
22. Das S, Mahapatra SK, Tripathy S, Chattopadhyay S, Dash SK, Mandal D, Das B, Hati AK, Roy S, 2014. Double mutation in the *pfmdr1* gene is associated with emergence of chloroquine-resistant *Plasmodium falciparum* malaria in eastern India. *Antimicrob Agents Chemother 58:* 5909–5915.
23. Meng H, Zhang R, Yang H, Fan Q, Su X, Miao J, Cui L, Yang Z, 2010. In vitro sensitivity of *Plasmodium falciparum* clinical isolates from the China–Myanmar border area to quinine and association with polymorphism in the Na^+/H^+ exchanger. *Antimicrob Agents Chemother 54:* 4306–4313.
24. Wang Z, Parker D, Meng H, Wu L, Li J, Zhao Z, Zhang R, Fan Q, Wang H, Cui L, Yang Z, 2012. In vitro sensitivity of *Plasmodium falciparum* from China–Myanmar border area to major ACT drugs and polymorphisms in potential target genes. *PLoS One 7:* e30927.
25. Hao M, Jia D, Li Q, He Y, Yuan L, Xu S, Chen K, Wu J, Shen L, Sun L, Zhao H, Yang Z, Cui L, 2013. In vitro sensitivities of *Plasmodium falciparum* isolates from the China–Myanmar border to piperaquine and association with polymorphisms in candidate genes. *Antimicrob Agents Chemother 57:* 1723–1729.
26. Wong RP, Lautu D, Tavul L, Hackett SL, Siba P, Karunajeewa HA, Ilett KF, Mueller I, Davis TM, 2006. In vitro sensitivity of *Plasmodium falciparum* to conventional and novel antimalarial drugs in Papua New Guinea. *Trop Med Int Health 15:* 342–349.
27. Daniels R, Ndiaye D, Wall M, McKinney J, Sene PD, Sabeti PC, Volkman SK, Mboup S, Wirth DF, 2012. Rapid, field-deployable method for genotyping and discovery of single-nucleotide polymorphisms associated with drug resistance in *Plasmodium falciparum*. *Antimicrob Agents Chemother 56:* 2976–2986.
28. Francis D, Nsobya SL, Talisuna A, Yeka A, Kamya MR, Machekano R, Dokomajilar C, Rosenthal PJ, Dorsey G, 2006. Geographic differences in antimalarial drug efficacy in Uganda are explained by differences in endemicity and not by

known molecular markers of drug resistance. *J Infect Dis 193:* 978–986.

29. Dokomajilar C, Nsobya SL, Greenhouse B, Rosenthal PJ, Dorsey G, 2006. Selection of *Plasmodium falciparum pfmdr1* alleles following therapy with artemether-lumefantrine in an area of Uganda where malaria is highly endemic. *Antimicrob Agents Chemother 50:* 1893–1895.
30. Mbogo GW, Nankoberanyi S, Tukwasibwe S, Baliraine FN, Nsobya SL, Conrad MD, Arinaitwe E, Kamya M, Tappero J, Staedke SG, Dorsey G, Greenhouse B, Rosenthal PJ, 2014. Temporal changes in prevalence of molecular markers mediating antimalarial drug resistance in a high malaria transmission setting in Uganda. *Am J Trop Med Hyg 91:* 54–61.
31. Mohapatra PK, Sarma DK, Prakash A, Bora K, Ahmed MA, Sarma B, Goswami BK, Bhattacharyya DR, Mahanta J, 2014. Molecular evidence of increased resistance to anti-folate drugs in *Plasmodium falciparum* in north-east India: a signal for potential failure of artemisinin plus sulphadoxine-pyrimethamine combination therapy. *PLoS One 9:* e105562.
32. Mixson-Hayden T, Jain V, McCollum AM, Poe A, Nagpal AC, Dash AP, Stiles JK, Udhayakumar V, Singh N, 2010. Evidence of selective sweeps in genes conferring resistance to chloroquine and pyrimethamine in *Plasmodium falciparum* isolates in India. *Antimicrob Agents Chemother 54:* 997–1006.
33. Das MK, Lumb V, Mittra P, Singh SS, Dash AP, Sharma YD, 2010. High chloroquine treatment failure rates and predominance of mutant genotypes associated with chloroquine and antifolate resistance among falciparum malaria patients from the island of Car Nicobar, India. *J Antimicrob Chemother 65:* 1258–1261.
34. Ndiaye D, Dieye B, Ndiaye YD, Van Tyne D, Daniels R, Bei AK, Mbaye A, Valim C, Lukens A, Mboup S, Ndir O, Wirth DF, Volkman S, 2013. Polymorphism in *dhfr*/*dhps* genes, parasite density and ex vivo response to pyrimethamine in *Plasmodium falciparum* malaria parasites in Thies, Senegal. *Int J Parasitol Drugs Drug Resist 3:* 135–142.
35. Mharakurwa S, Kumwenda T, Mkulama MA, Musapa M, Chishimba S, Shiff CJ, Sullivan DJ, Thuma PE, Liu K, Agre P, 2011. Malaria antifolate resistance with contrasting *Plasmodium falciparum* dihydrofolate reductase (DHFR) polymorphisms in humans and *Anopheles* mosquitoes. *Proc Natl Acad Sci USA 108:* 18796–18801.
36. Mkulama MA, Chishimba S, Sikalima J, Rouse P, Thuma PE, Mharakurwa S, 2008. Escalating *Plasmodium falciparum* antifolate drug resistance mutations in Macha, rural Zambia. *Malar J 7:* 87.
37. Dorsey G, Dokomajilar C, Kiggundu M, Staedke SG, Kamya MR, Rosenthal PJ, 2004. Principal role of dihydropteroate synthase mutations in mediating resistance to sulfadoxine-pyrimethamine in single-drug and combination therapy of uncomplicated malaria in Uganda. *Am J Trop Med Hyg 71:* 758–763.
38. Ahmed A, Lumb V, Das MK, Dev V, Wajihullah, Sharma YD, 2006. Prevalence of mutations associated with higher levels of sulfadoxine-pyrimethamine resistance in *Plasmodium falciparum* isolates from Car Nicobar Island and Assam, India. *Antimicrob Agents Chemother 50:* 3934–3938.
39. Lumb V, Das MK, Mittra P, Ahmed A, Kumar M, Kaur P, Dash AP, Singh SS, Sharma YD, 2009. Emergence of an unusual sulfadoxine-pyrimethamine resistance pattern and a novel K540N mutation in dihydropteroate synthetase in *Plasmodium falciparum* isolates obtained from Car Nicobar Island, India, after the 2004 Tsunami. *J Infect Dis 199:* 1064–1073.
40. Yang Z, Li C, Miao M, Zhang Z, Sun X, Meng H, Li J, Fan Q, Cui L, 2011. Multidrug-resistant genotypes of *Plasmodium falciparum*, Myanmar. *Emerg Infect Dis 17:* 498–501.
